# Research advances on flotillins

**DOI:** 10.1186/1743-422X-8-479

**Published:** 2011-10-25

**Authors:** Feng Zhao, Jie Zhang, Yong-Sheng Liu, Li Li, Ya-Li He

**Affiliations:** 1 State Key Laboratory of Veterinary Etiological Biology, Lanzhou Veterinary Research Institute, Chinese Academy of Agricultural Sciences. Lanzhou, 730046, Gansu, P.R. China; 2 The People's Government of Huaikou Town in Jintang County of Chengdu City , Chengdu, 610404, Sichuan, P.R. China; 3 School of Public Health, Lanzhou university, Lanzhou, 730000, Gansu, P.R. China

**Keywords:** Flotillin-1, Flotillin-2, Lipid raft, Signal transduction, Prion protein, GPI-anchored proteins

## Abstract

The proteins of flotillin-1 and flotillin-2 were originally discovered in axon regeneration of goldfish retinal ganglion cells. They are generally used as marker proteins of lipid rafts and considered to be scaffolding proteins of lipid microdomains. Although they are ubiquitously expressed and well-conserved from fly to man, their exact functions remain controversial. In this review, we summarize the structure of flotillins and some functions of them, such as regulating axon regeneration, endocytosis, T cell activation, insulin signaling, membrane protein recruitment, roles in the progression of some diseases and so on.

## Introduction

Lipid rafts, which act as signaling and sorting platforms for numerous molecules, are stero- and sphingolipid-enriched small microdomains of cell membrane [1,2]. Flotillins, also called reggies, are considered to be scaffolding proteins of lipid rafts and are generally used as marker proteins of lipid microdomains. There are two homologous members in flotillin proteins family, flotillin-1 and flotillin-2. These proteins were originally discovered as regeneration molecules upregulated in regenerating axons of goldsish retinal ganglion cells after lesion of the optic nerve and thus named reggies for regeneration [3]. Then, Bickel et al. found that they were insoluble in TritonX-100 and float after sucrose density centrifugation, and hence named flotillins [4]. Actually, reggie-1 corresponds to flotillin-2, while reggie-2 corresponds to flotillin-1.

Flotillins are evolutionarily well-conserved and ubiquitously expressed from fly to man [5]. The identity of amino acid sequence of flotillins among vertebrate is about 90%, and the identity between vertebrate and invertebrates is about 64% [6]. Flotillin-2 has a wide distribution in different tissues, while the expression of flotillin-1 seems to be more restricted in mammalian [7]. Flotillin-1 is expressed most abundantly in brain, heart, lung, placenta [8], and in hematopoietic cells [9]. The expression analysis of flotillin-2 in mouse and human tissues by quantitative PCR suggested that it could express in all tissues. In some cell types, flotillins also localize in endosomal compartments, phagosomes, Golgi compartment and even exosomes [10-13]. Unexpectedly, flotillin-1 can colocalize with PTOV1 in the nucleus [14]. The human flotillin-1 gene is located on chromosome 6p21.3, whereas the human flotillin-2 gene is located on chromosome 17q11-12. Both genes are single-copy gene consisting of 13 and 11 exons, respectively, and both code a protein of 47 kDa [8,15].

### Structure of flotillins

Flotillins are considered to belong to the SPFH (stomatin, prohibitin, flotillin, HflK/C) protein family, which share a novel homology at their N-terminus region [16]. Moreover, their C-terminal region has several short repeat motifs called flotillin repeats, which are important for the formation of homo- and hetero-oligomers [17]. Flotillin proteins do not traverse the membrane but associate with some other proteins resided at the other side of them [18]. Thus, a transmembrane protein may exist which mediate the association of them. Flotillin-1 contains a palmitoylation site in Cys34, which is considered to be essential for the plasma membrane localization of it in kidney cells [19]. However, the palmitoylation site in Cys34 seems to be not critical on the localization of flotillin-1 in adipocytes [20]. Two hydrophobic stretches (amino acids 10-36 and 134-151) in flotillin-1 can facilitate its membrane association, especially the first one is more important [20]. Thus, the membrane targeting signals of flotillin-1 may be dependent on cell types. Unlike flotillin-1, flotillin-2 is associated with membranes through myristoylation in Gly2 and manifold palmitoylation (Cys4, Cys19 and Cys20) [21]. The stimulation of cells by epidermal growth factor (EGF) leads to a Tyr163-dependent translocation of flotillin-2 from the plasma membrane into endosomes [22].

Both flotillins are palmitoylated in the hetero-oligomers of flotillin-1 and flotillin-2, and the fully acylated hetero-oligomers on a cytoplasmis vesicle ultimately fuses with the plasma membrane [13]. The fully acylated hetero-oligomers can be phosphorylated by Fyn, then they were endocytosed at the plasma membrane [23,24]. They can be transferred from a early endosome to the late endosomal compartment [24,25]. Flotillins were originally considered to reside in plasma membrane caveolae and form hetero-oligomers with caveolar proteins [7]. This view was contradictory to the notion that flotillins are localized to non-caveolar microdomains [21,26].

### Functions of flotillins proteins

Although flotillins are evolutionarily conserved and universally expressed, their functions have remained controversial. Here, we review the recent findings providing novel insights into the function of flotillins. Both flotillins are preferentially associated with each other in hetero-oligomeric complexes, forms membrane microdomains that participate many cellular activities. The expression of one is regulated by the other, and reduction in one causes the reduction of the other [23]. They are involved in axon regeneration and neuronal differentiation, endocytosis, T-lymphocyte activation, membrane protein recruitment and so on. Furthermore, flotillin-1 also plays an important role in insulin signaling and cell proliferation, while flotillin-2 may influence tumor progression.

### Axon regeneration and neuronal differentiation

The flotillin proteins were originally discovered in neurons during axon regenetation, implying that they may be involved in regeneration of axon. During axon regeneration, flotillins were upregulated in retinal ganglion cells (RGC) of fish [3]. Munderloh et al. found that downregulation of flotillins triggered a clear reduction (up to 70%) of the number of regenerating axon in zebrafish, indicating that flotillins are indeed necessary for axon regeneration [27]. Furthermore, they are upregulated in mammalian retinal ganglion cells [28]. The expression levels of flotillins were downregulated in mammalian hippocampal neurons, causing the neurons failed to differentiate [29]. The knockdown of flotillins by flotillin-specific siRNAs restrained the axon regeneration and differentiation in hippocampal and N2a neurons [27]. Flotillins were upregulated during neutrophilic differentiation of HL-60 cells [30]. Additionally, Langhorst reported that expression of a trans-negative flotillin-2 deletion mutant, which interferred the oligomerization of flotillin, could inhibit insulin-like growth factor (IGF)-induced neurite outgrowth in N2a cells and impair differentiation of primary rat hippocampal neurons in vitro [25]. Taken together, flotillins clearly regulate regeneration and neuronal differentiation.

### Endocytosis

The association between flotillins and Src family kinase Fyn has been reported by several studies. In adipocytes, T lymphocytes and neurons, flotillins may interact with Fyn, indicating that flotillins have important roles in the formation of signal transduction centers [13,20]. Epidermal growth factor (EGF)-induced endocytosis depended on the phosphorylation of tyrosine residues on Tyr-160 in flotillin-1 and Tyr-163 in flotillin-2 by Fyn, and mutation of these two residues to phenylalanine prevents Fyn-induced flotillin internalisation [24,22]. Aït-Slimane found that the down-expression of flotillin-2 inhibited endocytosis of glycosyl phosphatidylinositol (GPI)-anchored proteins in hepatic cells, suggesting that endocytosis of GPI-anchored proteins depend on a clathrin- and flotillin-dependent pathway [31]. Cremona et al. reported that the flotillin-1-enriched membrane microdomains were required in protein kinase C (PKC)-triggered dopamine transporter (DAT) endocytosis [32]. Liang et al. suggested that flotillins play an important role in Niemann-Pick C1-like 1 (NPC1L1)-mediated cholesterol uptake. Knockdown of flotillins notablely attenuated the uptake of cholesterol and endocytosis of NPC1L1 [33].

### Insulin signaling

The exact role of flotillin-1 has been characterized in molecular detail in case of insulin signaling. Baumann et al. found that flotillin-1 formed a ternary complex with Cb1 and Cb1-associated protein (CAP) by yeast two-hybrid screening [34]. This complex was recruited into lipid rafts after insulin stimulation through interaction between the first hydrophobic domain of flotillin-1 and the SoHo (sorbin homology) domain of CAP [20,34]. Prohibiting this step will block the uptake of glucose by the glucose transporter type-4 GLUT4 [34]. However, some studies suggested that the role of flotillin-1 in insulin signaling might be independent of the interaction with CAP [35,36]. The phosphorylation of Cb1 recruites the CrkⅡ-C3G complex to lipid rafts, then C3G specifically activates the small guanosine 5'-triphosphate-hydrolyzing enzyme (GTPase) TC10 [37]. The activation of TC10 is crucial for insulin-stimulated glucose uptake and translocation of GLUT4 from internal storage sites to the cell surface [37]. Fecchi et al. demonstrated that GLUT4 localizes in perinuclear regions with flotillin-1 in skeletal muscle cells. GLUT4 moves to the sarcolemma where uptake case of glucose occurs when insulin stimulation. However, if flotillin-1-based domains are interferred by a cholesterol-sequestering agent, insulin can not stimulate GLUT4 translocation and uptake of glucose [38].

### Function in cell proliferation

As stated before, flotillin-1 can translocate into the nucleus. Flotillin-1 interacts with the mitogenic protein PTOV1, which shuttles between the nucleus and the cytoplasm in PC-3 and COS-7 cells. Flotillin-1 entered the nucleus following PTOV1 during S phase[14]. Depletion of either flotillin-1 or PTOV1 both could inhibit cell proliferation observably, while over-expression of either protein strongly induced proliferation [39]. Gómez et al. reported that flotillin-1 was crucial for maintaining the levels of the mitotic regulator Aurora B, and interacted with Aurora B directly through its SPFH domain. Flotillin-1 increased Aurora B levels and activity when translocated into the nucleus, while depletion of flotillin-1 downregulated the levels and activity of Aurora B [40].

### Platform function for signaling in T cells

In T cells, flotillin microdomains are pre-clustered at one pole of the cell to form flotillin cap, which is important for T cell activation [9]. When T cell was stimulated, signaling molecules, including Thy-1, TCR/CD3, Fyn, lck and LAT, are recruited to the stable flotillin caps [41]. Antibody crosslinking of GPI-anchored proteins Thy-1 or PrP^c ^(cellular prion protein) also leads to selective association with the cap [42]. These signaling molecules are involved in the activation of T cell. The trans-negative flotillin-2 deletion mutant, which interferes with assembly of flotillin cap, blocked raft polarization and macrodomain formation after T cell activation and led to the incorrect positioning of the guanine nucleotide exchange factor Vav, resulting in defects in cytosbeletal reorganization [41].

PrP^c^, which is activated in the flotillin cap, induces the phosphorylation of the mitogen-activated protein (MAP) kinase ERK1/2 and elicits a distinct Ca^2+ ^signal resulting in recruitment of the major T cell receptor component CD3 although the recruitment is not sufficient [43]. The recruitment of T cell receptor encompasses communication with Src tyrosine kinases (Fyn, Lck, Src), Rho-GTPase and the signaling molecule Vav [41]. Therefore, the microdomains of flotillin provide a platform for signaling in T cells.

### Role in tumor progression

Hazarika et al. found that both flotillin-2 protein and mRNA were increased in tumorigenic and metastatic melanoma cell lines in vitro. SB2 melanoma cells altered to highly tumorigenic and metastatic in nude mice after transfection of flotillin-2. These cells also proliferated fast in the absence of serum, and thrombin enhanced their migration. Furthermore, the expression of protease activated receptor 1 (PAR-1) mRNA increased in these cell [44]. PAR-1 is a transmembrane, G-protein-coupled receptor involved in melanoma progression. In contrast, depletion of flotillin-2 by means of specific small-interfering RNAs made substantially less flotillin-2 and PAR-1 mRNA [44]. Thus, flotillin-2 may play an important role in affecting tumor progression through interacting with PAR-1.

### Association with other proteins

Kato et al. suggested that flotillin-1 was a crucial molecule in IgE receptor-mediated mast cell activation, and involved in the activation of Lyn [45]. The interaction between flotillin-1 with CAP, Vinexin α and ArgBP2, respectively, all of which are members of the SoHo family, indicates that flotillin-1 related to the organization of the actin cytoskeleton [46]. The interaction between flotillin-1 and neuroglobin (Ngb) identified by GST-pulldown assays implies that flotillin-1 might recruit Ngb to lipid rafts as a means of preventing neuronal death [47]. Flotillin-2 interacts with F-actin through its SPFH domain and regulates its lateral mobility at the plasma membrane [48]. Flotillin-2 was found to interact with kinesin KIF9 and knockdown of flotillins reduced matrix degradation by pososomes, which suggest that flotillin and KIF9 proteins can regulate matrix degradation by macrophage podosomes [49]. Flotillin microdomains associate with several cytoskeletal proteins, particularly myosin Ⅱa and spectrin, suggesting that flotillins play an important roles during neutrophil migration in uropod formation and in the regulation of myosin Ⅱa [50]. The N-terminal portion of flotillins is crucial for its interaction with the heterotrimeric G protein α q subunit (Gαq). The knockdown of flotillins, especially flotillin-2, damped the UTP-induced activation of p38 mitogen-activated protein kinase (MAPK). The activation of p38 MAPK was inhibited by the Src family kinases, suggesting that flotillins modulated Gq-induced p38 MAPK activation [51].

### Flotillins in neurodegenerative diseases

Some studies suggested that flotillins play a role in the pathogenesis of neurodegenerative diseases such as prion diseases (BSE, scrapie and CJD et al.), Parkinson's and Alzheimer's diseases (AD). Prion diseases are caused by misfolding of cellular prion protein (PrP^c^). Stuermer et al. shown that PrP^c ^was closely associated with flotillins at the plasma membrane in lymphocytes. Moreover, cross-linking of PrP^c ^resulted in its clustering in the region of the preformed flotillin cap [42]. Flotillins were also found in lipid-rich vesicles from Jurkat T cells together with PrP^c ^[52]. Furthermore, scrapie prion protein (PrP^Sc^) is localized in flotillin-1 positive late endosomes in the central nervous system cells [53]. Thus, clustering of PrP may contribute to the spreading of prion diseases. The expression of flotillin-1 was upregulation in the substantia nigra of Parkinson's patients [54]. Rajendran et al. reported that cellular amyloid beta-protein (Aβ), which is a pathological hallmark of AD, accumulated in flotillin-1 positive endocytic vesicles. Additionally, flotillin-1 associated with extracellular Aβ plaques in AD patient brain sections [55]. Sratins, which strongly reduced the Aβ load by modulating the processing of the amyloid beta precursor protein and reduced the prevalence of AD, also reduced the expression of flotillin-1 [56]. These may indicate an association of flotillin-1 with AD. Taken together, these studies indicated that flotillins may participate in the progression of some neurodegenerative diseases.

## Conclusions

No matter what the details of the functions of flotillins be, it is obvious that flotillins do not simply function as scaffolding proteins for lipid rafts but active signaling partners taking part in various vital cell processes. It involves in axon regeneration and neuronal differentiation, endocytosis, T-lymphocyte activation, etc. Moreover, a hypothesis suggests that flotillins are involved in the recruitment of membrane and specific membrane proteins to specific regions or domains of the cell [29]. Some reports shown that flotillins are involved in the recruitment of the GLUT4 from internal stores to the plasma membrane in adipocytes [34,57], in the recruitment of cadherin to the cell membrane of embryonic and epithelial cells, in the recruitment of the TCR to the T cell cap in lymphocytes, and in the recruitment of integrins to focal adhesions [58]. This function is required in all cells which explains why flotillins are expressed in many or all cells in invertebrates and vertebrates. The microdomains of flotillins may facilitate the assembly of special cell surface proteins and signaling complexes at the plasma membrane in a cell type specific manner, hence causing the internal proteins recruiting to the membrane domains and also involving some signaling processes. Now that flotillins are ubiquitously expressed in so many cells, they are surely involved in some more functions excepting we have found. So there is a wide prospect about the research of flotillins. However, the exact functions of flotillins remain controversial and further experiment must be supported in many aspects.

## Abbreviations

PTOV1: prostate tumor overexpressed gene 1 protein; EGF: epidermal growth factor; RGC: retinal ganglion cells; IGF: insulin-like growth factor; GPI: glycosyl phosphatidylinositol; PKC: protein kinase C; DAT: dopamine transporter; NPC1L1: Niemann-Pick C1-like 1; CAP: Cb1-associated protein; SoHo: sorbin homology; GLUT4: glucose transporter type-4; Crk: CT10-related kinase; GTPase: guanosine 5'-triphosphate-hydrolyzing enzyme; TCR: T cell receptor; LAT: linker of activated T cells; PrP^c^: cellular prion protein; MAP: mitogen-activated protein; ERK1/2: extracellular regulated kinases 1/2; PAR-1: protease activated receptor 1; Ngb: neuroglobin; Gαq: G protein α q subunit; MAPK: mitogen-activated protein kinase; BSE: bovine spongiform encephalopathy; CJD: Creutzfeldt-Jakob disease; AD: Alzheimer's diseases; PrP^Sc^: scrapie prion protein; Aβ: amyloid beta-protein.

## Competing interests

The authors declare that they have no competing interests.

## Authors' contributions

FZ, JZ and LL conceived of the study and drafted the manuscript; YLH helped to draft the manuscript. YSL supervised the research and helped to draft the manuscript. All authors read and approved the final manuscript.

## Authors' information

FZ: Qualification: Bachelor's degree. Current position: Post Graduate of Lanzhou Veterinary Research Institute, Chinese Academy of Agricultural Sciences (CAAS).

JZ: Qualification: Doctor's degree. Current position: As a researcher in the Department of Virology, Lanzhou Veterinary Research Institute, CAAS.

YSL: Qualification: Doctor's degree. Current position: As a associate professor in the Department of Virology, Lanzhou Veterinary Research Institute, CAAS.

LL: Qualification: Bachelor's degree. Current position: As a College-Graduate Village Official in the People's Government of Huaikou Town in Jintang County of Chengdu City

YLH: Qualification: Bachelor's degree. Current position: Post Graduate of School of Public Health, Lanzhou university.
